# Ultrasound-Assisted Synthesis of Glycerol Carbonate Using Potassium-Modified Silicalite-1 as a Catalyst

**DOI:** 10.3390/molecules30071590

**Published:** 2025-04-02

**Authors:** Jolanta Kowalska-Kuś, Ewa Janiszewska, Agnieszka Held, Aldona Jankowska, Anetta Hanć, Stanisław Kowalak

**Affiliations:** Faculty of Chemistry, Adam Mickiewicz University, Uniwersytetu Poznańskiego 8, 61-614 Poznań, Poland; jolakow@amu.edu.pl (J.K.-K.); awaclaw@amu.edu.pl (A.H.); aljan@amu.edu.pl (A.J.); anettak@amu.edu.pl (A.H.)

**Keywords:** glycerol carbonate, transesterification reaction, DMC, silicalite-1, basic sites, ultrasounds

## Abstract

This study investigates the use of potassium-modified silicalite-1 as a catalyst for the transesterification of glycerol to glycerol carbonate (Glyc. Carbonate) with dimethyl carbonate (DMC). Silicalite-1, typically inactive due to the absence of extra-framework cations, was modified with potassium compounds (fluoride, chloride, and hydroxide), which create basic sites by interacting with structural defects formed through silicon removal. This modification significantly enhances the catalyst’s performance in glycerol transesterification. The reaction was conducted in both conventional batch reactor and ultrasound-assisted systems, including an ultrasonic bath and an ultrasonic probe, either within the bath or directly in the reactor. The direct ultrasound probe application yielded the most remarkable results, achieving a 96% Glyc. Carbonate yield at 70 °C in just 15 min—dramatically surpassing the batch reactor, which reached approximately 5%. These findings highlight the synergistic effect of potassium modification and ultrasound-assisted transesterification, offering a highly efficient and sustainable approach for glycerol valorization.

## 1. Introduction

Over the past decade, a significant increase in the use of renewable energy sources has been observed worldwide. Among renewable alternatives, biodiesel has emerged as a promising replacement for petroleum-based diesel [1]. Produced through the transesterification of vegetable oils or animal fats with alcohol, biodiesel consists of fatty acid methyl esters (FAMEs) and is valued for its biodegradability, low toxicity, and reduced carbon emissions—up to 70% less than conventional diesel over its lifecycle [2]. These advantages have fueled a surge in biodiesel production worldwide, positioning it as a cornerstone of sustainable energy systems. Despite its benefits, biodiesel production poses challenges, particularly the effective utilization of its primary by-product, glycerol. For every 100 kg of biodiesel produced, approximately 10 kg of glycerol is generated [3,4]. This surplus has driven extensive research into expanding glycerol’s applications in both academic and industrial settings. As a versatile feedstock, glycerol offers immense potential for conversion into high-value chemicals [5,6,7]. Among its derivatives, glycerol carbonate (Glyc. Carbonate) has garnered significant attention due to its desirable attributes, including biodegradability, low toxicity, high boiling point, and chemical versatility. These properties make Glyc. Carbonate is a multifunctional compound with applications across diverse industries, such as green solvents, surfactants, personal care products, and energy storage solutions [8,9,10]. In addition to its role as a sustainable alternative in beauty and skin care products, Glyc. Carbonate serves as an electrolyte in lithium batteries, replacing traditional materials like propylene and ethylene carbonates [11]. It is also utilized in the production of gas separation membranes [12] and materials such as polyurethane foams, coatings, paints, and detergents [13]. Furthermore, Glyc. Carbonate acts as a precursor to glycidol, an important intermediate in polymer synthesis, solidifying its position as a critical component in sustainable chemical manufacturing [14].

**Scheme 1:**
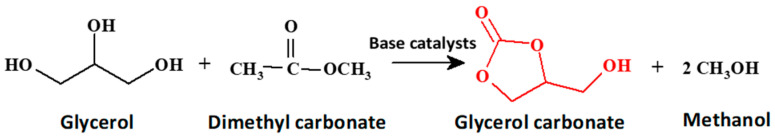
Pathway of the transesterification reaction of glycerol with dimethyl carbonate for the synthesis of glycerol carbonate.

Glycerol carbonate is commonly synthesized through the transesterification of glycerol with carbonation agents such as alkylene carbonates (e.g., ethylene or propylene carbonate), phosgene, or dialkyl carbonates (e.g., dimethyl or diethyl carbonate) [15]. Alkylene carbonates require reduced pressure for by-product separation, while phosgene’s toxicity limits its use [16,17]. Urea is another option but generates large amounts of ammonia, restricting industrial applicability [18]. Transesterification with dialkyl carbonates is the most promising method due to its non-toxic materials, mild conditions, and high yields (Scheme 1) [19,20]. This process, however, requires basic catalysts, and therefore, the search for stable, cost-effective, and sustainable catalysts remains key to advancing the commercial viability of this approach.

Among the most effective catalysts for this reaction are basic heterogeneous catalysts, which facilitate the transesterification process by promoting the reaction without the need for harsh reaction conditions [21]. These catalysts not only enhance the reaction efficiency but also offer the benefit of easy recovery and reuse, making them particularly suitable for large-scale, industrial applications. Several types of basic heterogeneous catalysts have been explored for glycerol transesterification, including alkaline earth metal oxides [22], mixed oxides [23,24], hydrotalcites [25], zeolites [26,27], and coal fly ash (CFA)-derived materials [28]. Alkaline earth metal oxides, such as calcium oxide (CaO) and magnesium oxide (MgO), are among the most widely studied for glycerol transesterification. CaO, with its strong basicity, provides high glycerol conversion rates due to its external surface basic sites, while MgO also exhibits good catalytic activity, which can be enhanced through modification with other materials like alumina [22,29]. Mixed oxide catalysts like calcium-lanthanum (Ca-La), zinc-lanthanum (Zn-La), and magnesium-aluminum-zirconium (Mg/Al/Zr) offer enhanced catalytic performance by combining the basicity of alkaline earth metals with the structural stability of transition metals [30]. Hydrotalcites, which are layered double hydroxides, exhibit inherent basic properties, and their catalytic activity can be further improved by modifying them with metal ions such as nickel or cobalt [31]. Among the heterogeneous systems studied for glycerol transesterification, zeolites have emerged as highly promising catalysts. Both natural forms (e.g., clinoptilolite) and metal-modified variants (e.g., sodium, potassium, or transition metals such as nickel) exhibit a combination of acidic and basic sites, enabling enhanced reaction rates and improved selectivity [20,26,27].

Most research on glycerol carbonate synthesis has identified basicity as the most critical factor in the transesterification reaction [32,33]. However, recent studies have also explored the influence of catalyst textural properties—such as pore volume, pore size, and the accessibility of active sites—on reaction performance [27,34,35,36]. According to Density Functional Theory (DFT) calculations [35], catalysts with pore sizes ranging from micropores to mesopores may enhance transesterification reactions, as the molecular diameter of glycerol carbonate is approximately 5.25 Å. When microporous catalysts such as zeolites were used, the glycerol transformation to glycerol carbonate was observed to occur either on their external surface or within their internal structure, depending on the zeolite’s shape and size. The literature reports that the catalytic activity of zeolites with small pore diameters of approximately 3 Å is primarily associated with their external surface. This is due to the diffusional restrictions imposed by smaller pores, which limit access to active sites and result in lower yields [27]. Additionally, studies on the crystallization kinetics of Na-LTA zeolites [37] have shown that the optimal conversion of glycerol to glycerol carbonate occurs in the early stages of crystallization. This is attributed to the open structure, which enhances catalytic performance by facilitating better diffusion of reactants to active sites. Further research [36] on calcium oxide supported by various porous materials has confirmed that mesoporous samples exhibit higher activity than microporous ones. These findings emphasize the significant role of textural properties, particularly pore volume, in glycerol transesterification to glycerol carbonate. Moreover, catalyst morphology and particle size have been reported as significant factors in glycerol conversion. Studies have demonstrated that both catalyst grinding [38] and the use of nanosheet-type catalysts can reduce diffusion limitations [34], providing easier access to active sites and thereby enhancing catalytic activity in glycerol transformation.

Conventionally, batch reactors that rely on traditional heating and mechanical stirring are used for synthesizing Glyc. Carbonate from glycerol. However, this method typically requires higher temperatures and longer reaction times to achieve high conversion rates. This limitation arises from restricted mass transfer and uneven energy distribution. Therefore, ultrasound-assisted reactors can address these challenges by utilizing cavitation to enhance mixing and mass transfer. Under ultrasonic irradiation, the liquid medium forms tiny bubbles that expand and contract during sound waves’ compression and rarefaction cycles. These bubbles suddenly expand and collapse violently, generating significant local heat and pressure. This intense energy promotes higher mass transfer and faster reaction rates [39]. As a result, ultrasonic irradiation is considered an eco-friendly alternative to conventional heating methods, offering high efficiency, mild reaction conditions, and no side reactions.

**Scheme 2:**
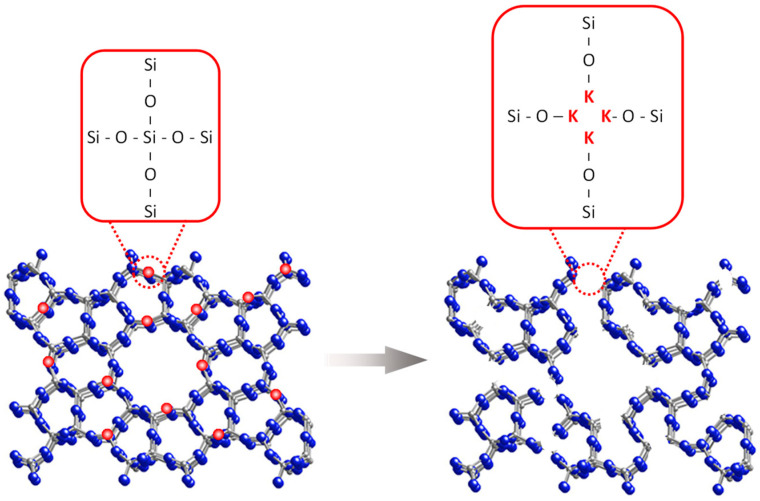
Modification of silicalite-1 with potassium fluoride/chloride/hydroxide.

Building on recent advances, this study investigates the use of commercially available silicalite-1, modified with alkali metal compounds such as potassium fluoride, chloride, and hydroxide, as a catalyst for glycerol transesterification under ultrasonic irradiation. To facilitate comparative analysis, reactions are performed in both a traditional batch reactor and ultrasound-assisted systems, including a conventional ultrasound bath and an ultrasound probe system. Silicalite-1, a silica-based material, lacks the intrinsic catalytic active sites typically found in zeolites, due to the absence of framework atoms like Al^3+^ [40]. This limitation has historically restricted its use as a catalyst. However, prior research indicates that modification with agents such as ammonium hydroxide, fluoride, or chloride can generate acidic properties, enhancing catalytic activity for reactions like glycerol-to-solketal transformation [41,42]. Extending this research, the present study further improves the catalytic performance of silicalite-1 by incorporating alkali metals. These metals interact with structural defects created by the selective removal of silicon atoms, generating basic sites crucial for catalytic activity (Scheme 2). By optimizing the basicity, textural properties of the catalysts, and the reaction conditions (using ultrasound), this study aims to provide an efficient solution for glycerol transesterification to glycerol carbonate. Thus, this work not only expands the catalytic potential of silicalite-1 but also highlights the synergy between catalyst modification and innovative reaction methods. The findings contribute to sustainable practices in biodiesel production, aligning with the principles of green chemistry by improving energy efficiency and minimizing waste in the glycerol valorization process.

## 2. Results and Discussion

### 2.1. Characterization of Catalysts

**Figure 1:**
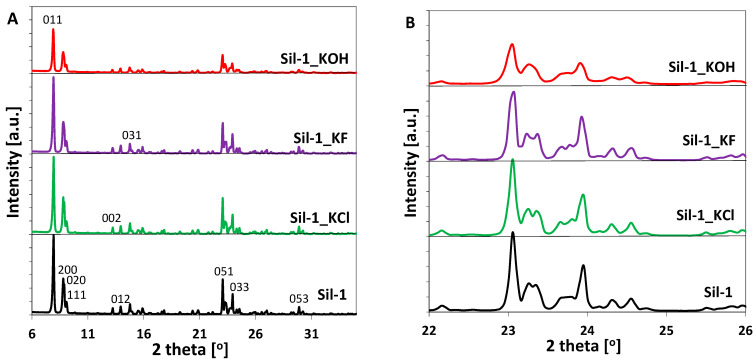
XRD patterns of silicalite-1 before and after modification with 1M solutions of various potassium compounds in a wide range (**A**) and in the range used for calculating the degree of crystallinity (**B**).

Silicalite-1 (Sil-1) was modified using 1M solutions of various potassium compounds, including KCl (Sil-1_KCl), KF (Sil-1_KF), and KOH (Sil-1_KOH). X-ray diffraction (XRD) measurements were conducted in the 6–60° 2Θ angle range to verify the structural correctness and assess the crystallinity of the obtained samples. The XRD patterns, presented in Figure 1, display only reflections characteristic of the MFI structure, confirming the correct structure of the initial sample and its preservation following treatment with potassium compound solutions [43]. The similar intensities of the reflections suggest comparable degrees of crystallinity among the majority of samples. Only the sample treated with KOH solution exhibits visible lower reflection intensities, indicating a reduction in crystallinity compared to the unmodified sample. This observation was corroborated by the relative XRD crystallinity, calculated as the ratio of the sum of the intensities of the most prominent reflections in the 23–25° range to the corresponding sum for the unmodified sample (Table 1, Figure 1B) [44]. Indeed, among the potassium compounds tested, KOH has the most pronounced effect, significantly decreasing crystallinity, while KF and KCl induce only minimal changes. Notably, the 1M KOH-treated sample exhibits a crystallinity of 55% relative to pristine Sil-1, highlighting the strong impact of hydroxide treatment.

**Figure 2:**
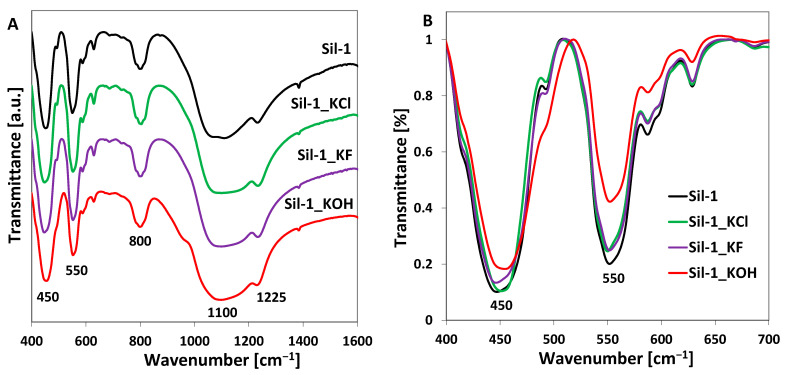
FTIR spectra of unmodified silicalite-1 and samples modified with 1M solutions of various potassium compounds in different wavenumber ranges: (**A**) 400–1600 cm^−1^ and (**B**) a magnified section of the spectra in the range of 400–700 cm^−1^.

Figure 2 depicts the FTIR spectra of the initial and modified samples. The spectra of the modified samples closely resemble that of the parent sample. The region between 1600 and 400 cm^−1^ in the spectra includes both structure-sensitive and structure-insensitive bands [45]. As noted by Göhlich et al. [46], the bands around 1100, 800, and 450 cm^−1^ correspond to the asymmetric, symmetric, and T–O bending vibrations of internal tetrahedra (T-Si atoms). These bands, being common to the IR spectra of silica and zeolites, are considered structure-insensitive. In contrast, the bands observed at 1225 cm^−1^ and 550 cm^−1^ are associated with external asymmetric vibrations and double five-ring (D5R) vibrations of tetrahedra in the MFI framework, respectively (Table S1). These structure-sensitive bands are particularly significant as they serve as indicators of the crystalline nature of the materials. The presence of these structure-sensitive bands in all the spectra confirms their crystallinity, consistent with the XRD findings. Furthermore, the presence of the characteristic band at 550 cm^−1^, corresponding to the double five-ring vibrations in MFI-type zeolites, confirms the preservation of the MFI framework during modification. Its reduced intensity in the modified samples suggests a decline in crystallinity (Figure 2B), as corroborated by the XRD patterns. The most pronounced decrease is observed for the Sil-1_KOH sample, aligning well with the XRD results.

**Figure 3:**
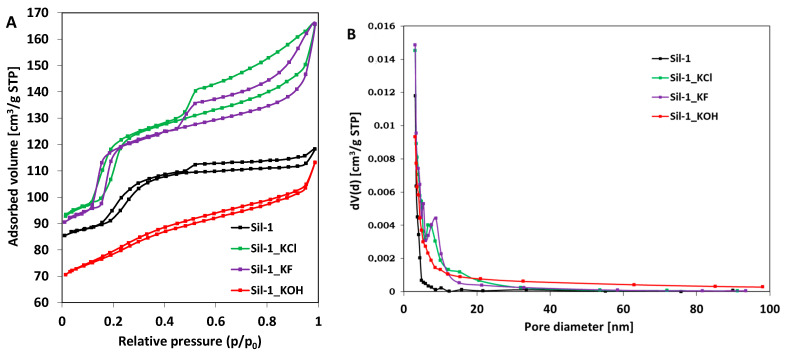
N_2_ adsorption/desorption isotherms (**A**) and pore size distribution (**B**) of unmodified silicalite-1 and samples modified with 1M solutions of different potassium compounds.

The influence of potassium-based modifications on the porosity of silicalite-1 was investigated using N_2_ adsorption/desorption measurements. The results are summarized in Table 1, while nitrogen adsorption/desorption isotherms and pore size distributions are presented in Figure 3. The parent silicalite-1 (Sil-1) exhibited both micropores and mesopores. The minor contribution of mesoporosity in the unmodified sample (S_ext_ = 42 m^2^/g, V_meso_ = 0.07 cm^3^/g) can be attributed to interparticle voids formed due to crystal agglomeration [47]. The type of potassium compound used for modification influenced the porous structure of the modified samples. Generally, treatment with potassium compounds increased the specific surface area and pore volume due to a desilication process, leading to mesopores formation, as evidenced by higher S_ext_ and V_meso_ values for modified samples. However, the sample treated with 1M KOH was an exception, showing a decrease in surface area and pore volume compared to the unmodified sample. This decline is attributed to a substantial reduction in microporosity (S_micro_ = 160 m^2^/g, V_micro_ = 0.09 cm^3^/g) compared to the initial sample (S_micro_ = 251 m^2^/g, V_micro_ = 0.12 cm^3^/g). Moreover, the more pronounced structural degradation likely resulted from the aggressive desilication by 1M KOH, leading to a significant loss of crystallinity, as confirmed by XRD analysis (Figure 1A, Table 1). In contrast, treatments with 1M KCl and 1M KF caused only minor alterations in the porosity. The micropore volumes remained unchanged compared to the parent sample, while the mesopore surface areas increased to 72 m^2^/g and 96 m^2^/g for KCl- and KF-modified samples, respectively. This is likely due to the limited silicon removal, which instead increased the roughness of the crystal’s external surface, as confirmed by SEM analysis (see below). These treatments yielded approximately 92%, indicating low silicon dissolution compared to the 31% yield for the 1M KOH treatment.

The N_2_ adsorption isotherm of the parent Sil-1 sample corresponds to a type IV with two hysteresis loops (Figure 3A). The first hysteresis loop appears in the p/p_0_ range of 0.15–0.35. This feature is characteristic of well-crystalized high-silica and silica-based zeolites with an MFI structure and can be attributed to the transition of nitrogen molecules from a fluid-like to a crystal-like phase within the micropores [48,49,50]. The second hysteresis loop in the p/p_0_ range of 0.5–1.0 can be attributed to interparticle voids formed by the agglomeration of Sil-1 crystals [48,51]. The isotherms of potassium salts (KCl and KF)-modified Sil-1 samples exhibit a similar shape to the unmodified sample but show a more pronounced high-pressure hysteresis loop. This enhancement is associated with capillary condensation in newly formed mesopores. These findings are supported by the data in Table 1 and the pore size distribution profiles (Figure 3B), which reveal the presence of additional larger pores in the modified samples compared to the unmodified silicalite-1. In the case of the Sil-1_KOH sample, the low-pressure hysteresis loop disappears, likely due to extensive amorphization of the sample, which was confirmed by XRD analysis (Figure 1). The isotherm of Sil-1_KOH exhibits a broad hysteresis loop across a wide p/p_0_ range without a distinct plateau at saturation pressure, indicating capillary condensation of the adsorbate in mesopores and macropores [52]. The formation of meso- and macropores in Sil-1_KOH results from structural defects introduced during KOH treatment, as further confirmed by SEM micrographs (Figure S1). Moreover, unlike the other materials, Sil-1_KOH displays a wide pore size distribution (Figure 3B). Its pore distribution curve follows a smooth, continuous course without a clear boundary between micro-, meso-, and macropores, indicating a hierarchical porous structure. This interconnected network of pores enhances mass transport and provides better accessibility to active sites, which in turn improves diffusion rates and reduces transport limitations.

**Figure 4:**
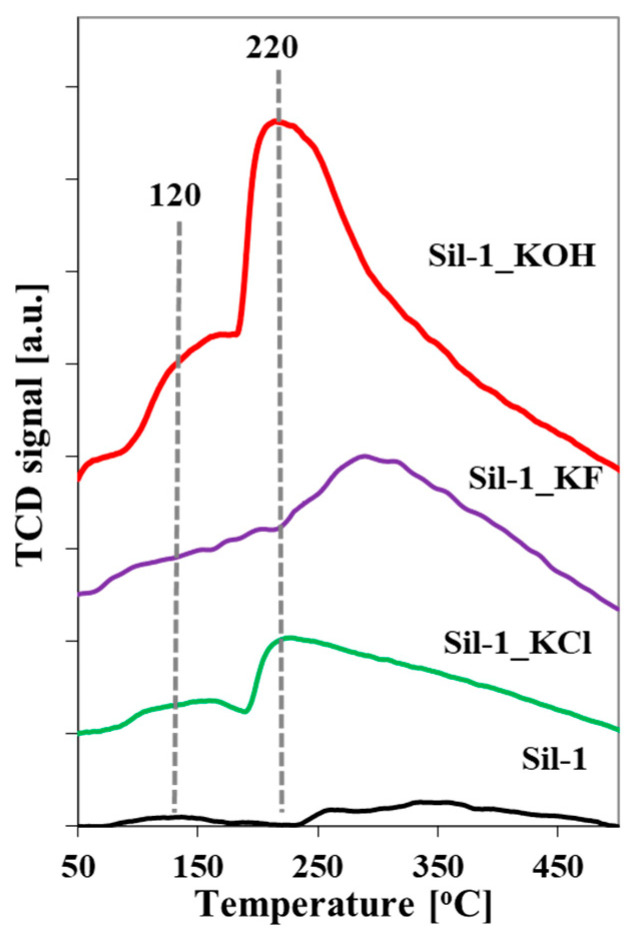
CO_2_-TPD profiles of unmodified silicalite-1 and samples modified with 1M solutions of various potassium compounds.

Both the strength and quantity of basic surface sites in the studied samples were determined using CO_2_-TPD measurements (Figure 4, Table 2). The initial silicalite-1 primarily exhibited CO_2_ desorption peaks at approximately 120 °C and 350 °C, corresponding to weak and strong basic sites, respectively. These basic sites originate from residual unwashed sodium atoms (confirmed by EDS analysis, see Table 3); however, their amount is negligible. Modification with potassium compounds enhanced the number of basic sites relative to the unmodified sample. As the potassium content increased, as estimated by EDS, the total number of basic sites exhibited a corresponding rise. For samples modified with potassium salts (KCl, KF), strong basic sites were predominant, whereas in the KOH-modified sample, moderate basic sites dominated. The difference in the proportion of moderate and strong basic sites was relatively small for KCl- and KF-modified samples, while for the KOH-modified sample, the predominance of moderate basic centers was much more pronounced. Among all the modified samples, the one treated with 1M KOH exhibited the highest number of basic sites.

**Figure 5:**
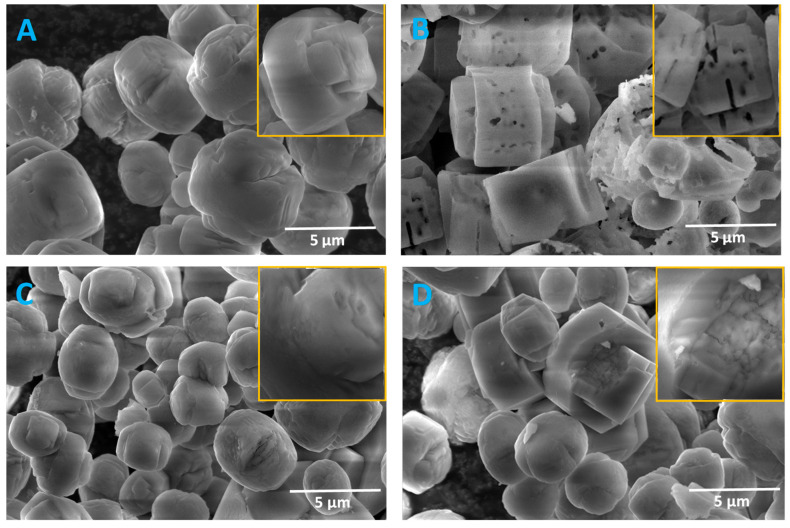
SEM micrographs of unmodified and K-modified silicalite-1 samples: Sil-1 (**A**), Sil-1_KOH (**B**), Sil-1_KCl (**C**), and Sil-1_KF (**D**).

The obtained materials were characterized by scanning electron microscopy (SEM) to investigate the morphology of the pristine material, evaluate the impact of the type of potassium compounds treatment on secondary porosity and surface morphology, and assess the structural stability of the zeolite framework. As illustrated in the accompanying micrographs (Figure 5), Sil-1 crystals exhibit a homogeneous morphology with a characteristic rounded shape, consistent with the previously reported literature [53]. Their well-defined crystals exhibit twin crystal growths on the (010) planes, which is characteristic of MFI-type zeolites (Figure 5A). The crystal surfaces are smooth and regular, devoid of visible defects or porosity, forming discrete, separated homogeneous entities. Treatment with potassium compounds led to noticeable morphological changes, including the formation of agglomerates, likely due to interactions between modified crystal surfaces (Figure 5B–D). The morphological evolution of hierarchical zeolites demonstrated a clear dependence on the type of potassium reagent used. Treatment with KF solution induced significant structural degradation, characterized by cracking and pronounced dissolution predominantly in the central regions of the crystals, where twin crystal intergrowths are located (Figure 5C). Exposure to KOH led to the formation of extensive secondary porosity across the entire crystal surface, with deep penetration into the crystalline framework (Figure 5B). Additionally, this modification caused substantial dissolution of the zeolite structure. Notably, despite the extensive etching, the sample treated with 1M KOH retained its crystallinity, as confirmed by X-ray diffraction (XRD) analysis (Figure 1A). Treatment with KCl exerted a minimal impact on crystal morphology, resulting in the least pronounced structural degradation among the tested conditions (Figure 5D). These findings highlight that the type of potassium source plays a pivotal role in controlling the extent of structural transformation and porosity development in zeolite crystals, with KOH treatments inducing the most extensive structural modifications.

Further confirmation of the effectiveness of potassium-compound modification is provided by the SEM-EDS analysis. Table 3 presents the elemental composition of both unmodified and K-modified silicalite-1 samples. In the parent silicalite-1, small amounts of Na were detected in addition to Si and O, likely as a residue from the synthesis process. Modification with potassium compounds led to a decrease in Si content due to silicon removal. However, the extent of Si removal varied depending on the potassium modifier used. The degree of silicon removal followed the order of Sil-1_KCl < Sil-1_KF < Sil-1_KOH. The decrease in silicon content (expressed as the Si/O ratio) indicates the formation of defects in the crystalline network. These defects facilitate the incorporation of extra-framework potassium cations, which correlates with an increase in potassium content and a simultaneous decrease in the Si/O ratio. These results are consistent with the observed structural modifications, which contributed to the formation of secondary porosity. The reduction in silicon content correlates with morphological changes, such as the formation of larger pores or structural damage (Table 1, Figure 5). Notably, the sample modified with KOH solution exhibited the greatest loss of silicon, while the KCl-modified sample showed the least.

**Figure 6:**
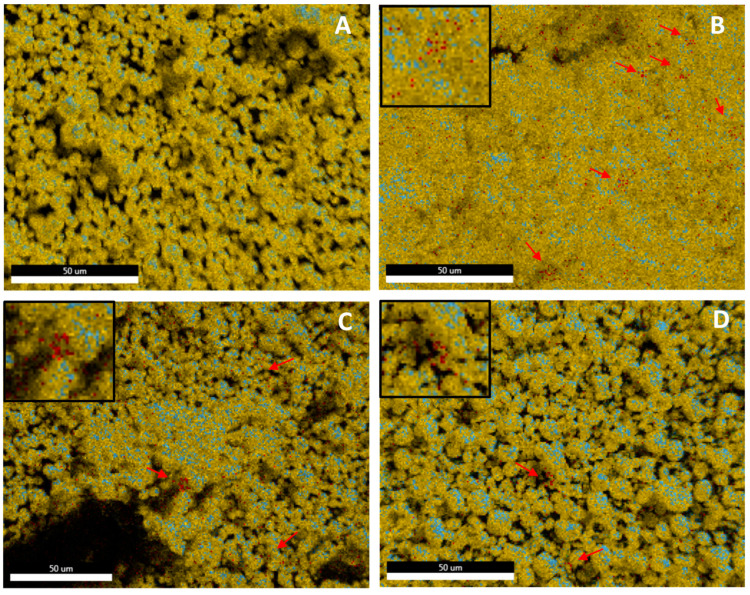
Elemental distribution map obtained by the EDS method for Sil-1 (**A**), Sil-1_KOH (**B**), Sil-1_KCl (**C**), and Sil-1_KF (**D**) samples. The colors represent the distribution of individual elements: Si (yellow), O (blue), and K (red).

Further elemental mapping indicates that, in the K-modified samples, the distribution of individual elements is heterogeneous, suggesting that alkali modification locally affects the sample’s surface (Figure 6). The EDS map analysis, combined with SEM images, shows that large clusters of potassium atoms are primarily located in regions where significant changes in crystal morphology occurred, such as the formation of larger pores. Additionally, the analysis did not detect fluorine or chlorine atoms, indicating their effective removal after the modification process.

### 2.2. Catalytic Activity in Batch Reactor

**Figure 7:**
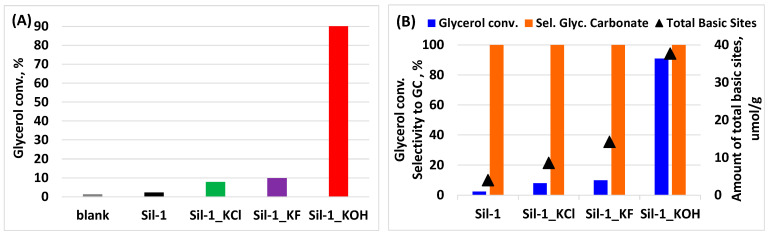
Glycerol conversion over as-synthesized and potassium-modified Sil-1 catalysts in glycerol carbonate synthesis (**A**); correlation between glycerol conversion, selectivity to glycerol carbonate, and the total number of basic sites (**B**). Reaction conditions: 10 wt.% catalyst, glycerol-to-DMC molar ratio of 1:5, 90 °C, 4 h.

The transesterification of glycerol with dimethyl carbonate (DMC) was carried out in a conventional batch reactor using a 10 wt.% catalyst and a glycerol-to-DMC molar ratio of 1:5. Unless stated otherwise, the reaction was conducted at 90 °C for 4 h. The catalytic performance results are presented in Figure 7A. The glycerol conversion over the Sil-1 sample was nearly identical to the blank test, barely exceeding 2%. When silicalite-1 was modified with potassium chloride (Sil-1_KCl) or potassium fluoride (Sil-1_KF), the glycerol conversion remained low, reaching only around 10%. Among the studied catalysts, Sil-1_KOH demonstrated the highest activity, achieving 91% glycerol conversion. Furthermore, glycerol carbonate was identified as the sole reaction product (Figure 7B).

As reported in previous studies, glycerol carbonate synthesis via glycerol transesterification occurs over basic catalysts [54]. Given the dependence of catalytic activity on catalyst basicity, the relationship between the number and strength of basic sites was investigated. As shown in Table 2, the total number of basic sites follows the order of Sil-1 < Sil-1_KCl < Sil-1_KF < Sil-1_KOH. This trend strongly correlates with glycerol conversion, which increases accordingly. All K-modified samples exhibited higher activity than the unmodified Sil-1 matrix, with glycerol conversion rising in line with the basicity of the tested materials (Table 2, Figure 7B). However, not only the number but also the strength of basic sites plays a crucial role in glycerol carbonate synthesis. To examine this effect, catalyst activity (expressed as glycerol conversion) was analyzed depending on the strength of basic sites in the studied samples. Among the tested materials, Sil-1_KOH exhibited the highest proportion of medium-strength basic sites (Table 2 and Figure S3) and achieved the highest glycerol conversion. These findings indicate that moderately strong basic sites are essential for efficient glycerol conversion. This observation aligns with literature reports [55], which identify medium-strength basic sites as the most favorable for the transesterification of glycerol with dimethyl carbonate to produce glycerol carbonate (Figure S3). In contrast, strong basic sites have been linked to the further conversion of glycerol carbonate into glycidol [27,55]. This conclusion is further supported by the R^2^ linear correlation factor (Table 2), which assesses the relationship between glycerol conversion and the number of different types of basic sites (total, low-temperature, medium-temperature, and high-temperature).

The significance of catalyst basicity in the transesterification reaction is further reinforced by the correlation between glycerol conversion and the density of basic sites. An increase in glycerol conversion was consistently accompanied by a rise in the density of basic sites, expressed in µmol/m² (Figure S4, Table S2).

Based on TPD-CO_2_ and EDS analysis, which confirmed the introduction of potassium ions with basic character, we proposed a mechanism in which potassium-containing basic sites are responsible for the surface-active species involved in the synthesis of glycerol carbonate (GC) (Scheme S1). The reaction initiates with the adsorption of glycerol on the catalyst surface, where the basic sites abstract a proton from glycerol, leading to the formation of a glyceroxide anion (C_3_H_7_O_3_^−^). In accordance with the Eley–Rideal mechanism, the glyceroxide anion remains adsorbed on the catalyst surface, while DMC remains in the bulk phase. The negatively charged glyceroxide anion interacts with the carbonyl oxygen of DMC, destabilizing the carbonyl group and enhancing the partial positive charge on the carbonyl carbon. This increase in electrophilicity facilitates a subsequent nucleophilic attack by the glyceroxide anion. This step results in the formation of methyl glyceryl carbonate (MGC) as an intermediate and the simultaneous release of a methoxide anion. The methoxide anion subsequently abstracts a proton from a basic site, generating methanol and restoring the catalyst’s active sites for further reactions. In the final step, MGC undergoes intramolecular cyclization. The oxygen anion from the secondary hydroxyl group of MGC attacks the carbonyl carbon, leading to the formation of the cyclic structure of glycerol carbonate. This completes the reaction, with glycerol carbonate being released as the final product. The cooperative interaction between the potassium-induced basic sites and the reactants is essential for ensuring the efficient synthesis of glycerol carbonate.

However, the presence of potassium and the resulting increase in the number of basic sites were not the only factors determining catalytic activity. A higher number of generated defects, along with an increased proportion of mesoporous surface area, also contributed to enhanced performance. As the number of defects grew and mesoporosity increased, the amount of formed glycerol carbonate likewise rose. Notably, potassium-modified samples exhibited a higher proportion of mesoporous to microporous surface areas, facilitating the reaction by mitigating diffusion and mass transport limitations. The highest degree of mesoporosity was achieved for the Sil-1_KOH sample, which demonstrated the highest activity in the studied reaction, as reflected in its lowest S_micro_/S_ext_ ratio (Table 1).

This textural advantage further enhanced overall catalytic efficiency, reinforcing the strong correlation between catalytic activity and surface basic site density in K-modified Sil-1 catalysts. These findings highlight the crucial role of catalyst basicity, as well as the influence of porosity, in the transesterification reaction.

### 2.3. Comparison Between Ultrasound-Assisted Transesterification of Glycerol and Conventional Heating

In a further experiment aimed at enhancing glycerol carbonate synthesis, three different ultrasonic techniques were applied: an ultrasonic bath, and an ultrasonic probe placed either in the ultrasonic bath or directly in the reactor (Scheme S2). The experiments were conducted using unmodified Sil-1 and Sil-1_KOH materials, selected as representative samples due to their significant differences in activity observed in the batch reactor.

**Figure 8:**
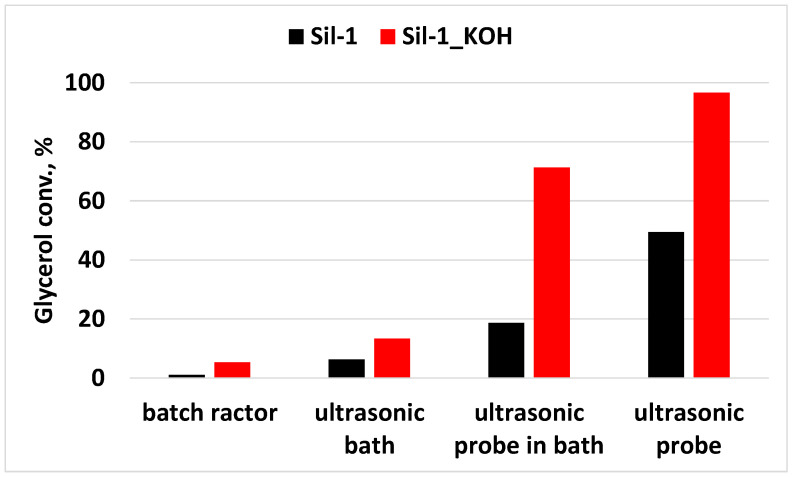
Effect of the applied procedures (batch reactor, ultrasonic bath, and ultrasonic probe in bath or ultrasonic probe) on glycerol conversion to glycerol carbonate using as-synthesized Sil-1 and potassium-modified Sil-1_KOH (reaction conditions: 10 wt.% of catalysts, glycerol-to-DMC molar ratio of 1:5, 70 °C, 15 min).

The reaction conditions closely resembled those used in conventional batch studies and were as follows: 10 wt.% catalyst, a glycerol-to-DMC molar ratio of 1:5, 70 °C, and a reaction time of 15 min. As observed, these experiments employed a lower temperature (70 °C instead of 90 °C) and a significantly shorter reaction time (15 min compared to the previously applied 4 h). These results clearly demonstrate the significant influence of the ultrasonic probe on glycerol conversion (Figure 8). In contrast, the effect of ultrasound-assisted methods using only an ultrasonic bath was minimal. A moderate improvement was observed when the ultrasonic probe was placed in the water bath. However, the most remarkable enhancement occurred when the ultrasonic probe was positioned directly inside the reactor. For the initially low-active Sil-1 material, glycerol conversion reached nearly 50%, while for the sample modified with 1M KOH, conversion was close to 100%. These findings confirm the positive impact of ultrasonic probe application in glycerol carbonate synthesis, enabling significantly improved catalytic performance at a lower temperature and in a much shorter reaction time compared to the conventional batch system. The observed improvements can be attributed to ultrasound-induced reductions in activation energy, enhanced mass transfer, and improved phase interaction [39]. Regarding the hypothesis that ultrasound promotes the reaction primarily through thermal catalysis, our findings suggest that localized heating does contribute to reaction kinetics. The approximately 50% conversion observed for the Sil-1 sample supports this notion. However, while thermal effects play a role, the primary driving forces behind the enhanced conversion are improved mass transfer and better catalyst dispersion.

Additionally, the glycerol conversion, expressed in terms of turnover frequency (TOF), which normalizes activity to a single active site per time unit, clearly demonstrates the beneficial effect of ultrasound on the synthesis of glycerol carbonate (Table 4). The TOF value for the Sil-1_KOH catalyst shows a significant enhancement when subjected to ultrasonic bath conditions, with the value doubling compared to conventional batch reactor conditions. Particularly noteworthy is the increase in TOF observed when using an ultrasonic probe in the bath, where the TOF value rises nearly 13-fold relative to the batch reactor. Furthermore, when the ultrasonic probe is in direct contact with the reaction mixture, the TOF increases even further, reaching approximately 17 times the value obtained in the batch reactor. These results suggest that ultrasound-assisted methods, particularly with direct probe contact, significantly boost catalytic efficiency, likely due to enhanced mass transfer, localized energy input, and intensified reaction conditions, all of which contribute to the substantial increase in glycerol conversion and the overall reaction rate (Figure S5).

## 3. Conclusions

This study investigates the use of potassium-modified silicalite-1 as a catalyst for the transesterification of glycerol to glycerol carbonate (Glyc. Carbonate) with dimethyl carbonate (DMC). Modification with different potassium compounds (fluoride, chloride, and hydroxide) resulted in different amounts of silicon removal and varying extents of potassium incorporation, which affected both the morphology and porosity of the catalyst. These morphological changes were confirmed by SEM micrographs, which showed distinct differences in the structure of the modified samples. The most significant effect on both basicity and morphology was observed in the KOH-treated sample, which exhibited the highest potassium content and basicity. The KOH modification also led to the greatest degree of silicon removal, resulting in the formation of secondary porosity, which was accompanied by a significant reduction in crystallinity.

The activity was strongly dependent on the catalyst’s basicity, with the KOH-modified sample demonstrating the highest contribution of basic sites, particularly medium-strength sites, which were found to be the most effective for glycerol transesterification with dimethyl carbonate. The reaction was conducted in both conventional batch reactors and ultrasound-assisted systems, including an ultrasonic bath and ultrasonic probe. The direct ultrasound probe application produced exceptional results for Sil-1_KOH, achieving a 96% Glyc. Carbonate yield at 70 °C in just 15 min—significantly outperforming the batch reactor, which reached only 5% conversion. These findings highlight the synergistic effect of potassium modification and ultrasound-assisted transesterification, offering a highly efficient and sustainable approach for glycerol valorization. By enhancing glycerol conversion and reducing reaction time, this method presents a promising, scalable solution for the efficient production of glycerol carbonate, with a strong emphasis on the importance of catalyst basicity, the role of medium-strength basic sites, and the influence of morphological changes in optimizing glycerol conversion.

## 4. Materials and Methods

### 4.1. Synthesis of Silicalite-1

In the preparation of silicalite-1, tetrapropylammonium bromide (TPABr, 98%, Sigma-Aldrich, St. Louis, MO, USA) and water glass (Na_2_O/SiO_2_/H_2_O = 11.1/27.9/60.8, Chempur, Piekary Śląskie, Poland) were used as the template and silicon source, respectively. The molar composition of the synthesis gel was 0.08 TPABr/1 SiO_2_/20 H_2_O. The 1.49 g of template (TPABr) was dissolved in 41.25 mL of distilled water and then combined with 10.24 mL of water glass while stirring. Phosphoric acid (~0.1 mL, 85%, POCh, Gliwice, Poland) was added dropwise to adjust the initial pH of the gel to a value of 11. The resulting reaction mixture was stirred for 0.5 h and then transferred to a Teflon-lined stainless-steel autoclave. The gel was crystallized at 170 °C under autogenous pressure for 22 h. The obtained product was filtered, washed with distilled water to neutral pH, dried at 100 °C, and calcined in air at 550 °C for 5 h to remove the residual template (with a heating rate of 2 °C per minute).

### 4.2. Modification of Silicalite-1 with Potassium Compounds Solutions

Silicalite-1 (designated as Sil-1) was modified using 1M solutions of salts and potassium hydroxide (KCl, KF, KOH). Before modification, silicalite-1 was calcined in a furnace for 2 h at 550 °C to obtain an anhydrous zeolite by removing water. In a round-bottom flask, 1 g of activated silicalite-1 was combined with 100 cm^3^ of the appropriate salt or hydroxide solution. The flask was then placed in an oil bath under a reflux condenser and heated at 60 °C for 1 h with stirring at 300 rpm. After this period, the mixture was filtered (without washing) and dried in air at room temperature. The dried Sil-1 from the first stage was again mixed with 100 cm^3^ of the appropriate solution and stirred at 60 °C for 1 h. Following this, the modified Sil-1 was filtered, washed with deionized water, dried in air at room temperature, and calcined in a furnace at 350 °C for 2 h. The obtained samples were labeled as Sil-1_X, where “X” denotes the potassium compound used.

### 4.3. Characterization

X-ray diffraction (XRD) analysis of the obtained materials was performed using a Bruker AXS D8 Advance diffractometer (Bruker, Billerica, MA, USA) equipped with a CuKα radiation source and a nickel filter. Measurements were carried out within the 2θ range of 6–60°. The XRD analysis aimed to confirm the crystal structure of the modified materials. The structural integrity of the zeolitic materials was assessed by comparing the XRD patterns of the initial silicalite-1 with those of the modified silicalites. Additionally, this method allowed for the estimation of the catalysts’ degree of crystallinity based on the intensity of the diffraction peaks.

Low-temperature nitrogen adsorption/desorption measurements were performed using a Quantachrome NOVA 1000e apparatus (Quantachrome, Boynton Beach, FL, USA). Prior to measurement, the samples were degassed at 300 °C for 16 h. Adsorption and desorption isotherms were recorded at −196 °C. The specific surface area was determined using the BET method, whereas the external surface area and micropore volume were calculated by the t-plot method. The total volume of pores was assessed using the single-point model (at p/p_0_ = 0.99). The mesopore volume was calculated as a difference between the total and micropore volumes (V_tot_–V_micro_). The BJH pore size distributions were derived from the adsorption branch.

Infrared spectroscopy studies were conducted using a pellet composed of 1 mg of the tested material and 200 mg of potassium bromide on a BRUKER-TENSOR 27 spectrophotometer (Bruker, Billerica, MA, USA). Measurements were performed using the transmission technique in the wavenumber range of 4000–400 cm^−1^ with a resolution of 1 cm^−1^. This method complements XRD analysis and is useful for confirming the crystallinity and verifying the correct structure of the MFI framework.

Scanning electron microscopy (SEM) studies were conducted using a Quanta FEG 250 (FEI) microscope (Thermo Fisher Scientific, Eindhoven, Netherlands) under high vacuum conditions with a beam accelerating voltage of 10 kV. Energy dispersive spectroscopy (EDS) analyses were performed at the same voltage using an Octane SDD EDS detector (EDAX, Mahwah, NJ, USA).

Measurements of temperature-programmed desorption of carbon dioxide (TPD-CO_2_) were carried out on a Pulse ChemiSorb 2705 (Micromeritics, Norcross, GA, USA) instrument. Prior to the measurements, about 100 mg of the sample was pretreated in helium at 550 °C for 30 min, then cooled down to 100 °C, and afterward, saturated with carbon dioxide for 30 min. The physically adsorbed CO_2_ was removed by purging with a helium flow at 100 °C for 60 min, and then the TPD analysis was carried out. All TPD-CO_2_ profiles presented in this work were collected in the temperature range of 50–500 °C with a heating rate of 10° min^−1^ and normalized to the same sample weight (1 g).

### 4.4. Catalytic Activity in Transesterification Reaction of Glycerol with Dimethyl Carbonate

Typically, the transesterification of glycerol with dimethyl carbonate (DMC) was carried out in a batch reactor with stirring at 400 rpm at 90 °C for 4 h. Before the reaction, the catalysts were pre-activated in an oven under static air conditions at 550 °C for 2 h to remove any adsorbed water. The reaction mixture, consisting of 1 g of glycerol (11 mmol), 4.2 cm^3^ of DMC (55 mmol), and 0.1 g of the pre-activated catalyst, was placed in closed glass vials located in a reaction block on a magnetic stirrer to ensure precise temperature control (batch reactor). For comparison, ultrasound-assisted transesterification was also performed using two ultrasonic methods: an ultrasonic bath (Bandelin RK 100H, 320 W, 35 kHz, Bandelin, Berlin, Germany) and an ultrasonic probe (Sonoplus HD 2070, 70 W, 20 kHz, Bandelin, Berlin, Germany). The ultrasonic probe was used either in a water bath (ultrasonic probe in a bath) or applied directly to the reaction mixture (ultrasonic probe). These reactions were conducted at 70 °C for 15 min, constrained by the probe’s operational limits, while maintaining a glycerol-to-DMC (G:DMC) molar ratio of 1:5 and using 0.1 g of catalyst. In both ultrasonic methods, the catalyst pre-treatment followed the same protocol as in the batch system. In the ultrasonic bath method, the vials used were identical to those in the batch system and were placed in a water bath maintained at 70 °C. In the ultrasonic probe method standard closed glass vials, identical to those used in the batch system, were placed inside the water bath (70 °C), and the ultrasonic probe was submerged in a water bath to generate ultrasound. In the approach called the ultrasonic probe, a specialized type of glass vial was used, similar in size to the standard vials but equipped with a cover designed to ensure a tight seal while allowing direct insertion of the ultrasonic probe. After the reaction, all mixtures were cooled and homogenized by adding 1.5 cm^3^ of dimethylformamide (DMF), followed by filtration to separate the catalyst. The components of the post-reaction mixture were identified via mass spectrometry (Varian 4000 GC-MS, Agilent Technologies, Santa Clara, CA, USA, VF-5ms column) and showed the presence of glycerol carbonate, methanol, unreacted glycerol, DMC, and DMF. Typically, the quantitative analysis of the post-synthesis reaction mixture was performed using gas chromatography on a VARIAN CP-3800 chromatograph (Agilent Technologies, Santa Clara, CA, USA) equipped with a Flame Ionization Detector (FID) and a VF-5ms column. The details of the analysis, including the column program, catalyst chromatograms, and GC-MS analysis, are presented in the Supplementary Materials.

Catalyst performance, including glycerol conversion, selectivity to glycerol carbonate, and glycerol carbonate yield, was assessed using the formulas described in [27] and can be found in the Supplementary Materials.

The turnover frequency (TOF) was determined by multiplying the initial moles of glycerol, its conversion, and its molecular weight and then dividing by the catalyst mass and reaction time (h^−1^).

## Figures and Tables

**Table 1:** Textural properties of the unmodified silicalite-1 and samples modified with 1M solutions of various potassium compounds.

Sample	C_XRD_ ^1^ [%]	S_BET_ ^2^ [m^2^/g]	S_micro_ ^3^ [m^2^/g]	S_ext_ ^4^ [m^2^/g]	V_tot_ ^5^ [cm^3^/g]	V_micro_ ^6^ [cm^3^/g]	V_meso_ ^7^ [cm^3^/g]	S_micro_/S_ext_
Sil-1	100	293	251	42	0.19	0.12	0.07	6.0
Sil-1_KCl	99	322	250	72	0.24	0.12	0.12	3.5
Sil-1_KF	93	344	248	96	0.26	0.12	0.14	2.6
Sil-1_KOH	55	231	160	70	0.18	0.09	0.09	2.3

^1^ C_XRD_—relative crystallinity, calculated according to [37], ^2^ S_BET_—specific surface area, ^3^ S_micro_—micropore surface, ^4^ S_ext_—external surface, ^5^ V_tot_—total pore volume, ^6^ V_micro_—micropore volume, ^7^ V_meso_—mesopore volume.

**Table 2:** The number of basic sites evaluated by temperature-programmed desorption of CO_2_ of parent and K-modified silicalite-1 samples; linear correlation factor as a function of the number of basic sites with glycerol conversion.

Sample	Concentration of Basic Sites, μmol/g			
	LT-BS ^1^ T ≤ 200 °C	MT-BS ^2^ 200–350 °C	HT-BS ^3^ T ≥ 350 °C	Total
Sil-1	1.2	0.7	2.1	4.0
Sil-1_KCl	1.6	3.2	3.8	8.6
Sil-1_KF	2.6	5.5	6.3	14.2
Sil-1_KOH	4.3	27.2	6.2	37.7
Glycerol conv. R^2^ factor ^4^	0.8652	0.992	0.3414	0.9558

^1^ LT-BS—the number of low-temperature basic sites; ^2^ MT-BS—the number of medium-temperature basic sites; ^3^ HT-BS—the number of high-temperature basic sites; ^4^ R^2^ linear correlation factor as a function of the number of each type of basic site (total, low-temperature, medium-temperature, and high-temperature) with glycerol conversion (Figure S2).

**Table 3:** The composition of the investigated samples estimated by EDS.

Sample	Si [at. %]	O [at. %]	K [at. %]	Na [at. %]	Si/O Ratio
Sil-1	42.00	57.54	-	0.46	0.73
Sil-1_KCl	41.03	58.57	0.40	-	0.70
Sil-1_KF	38.40	60.88	0.72	-	0.63
Sil-1_KOH	35.83	61.10	3.07	-	0.58

**Table 4:** Glycerol conversion to obtain glycerol carbonate, expressed as TOF (turnover frequency).

Sample	Method	Reaction Conditions	Glycerol Conversion [%]	TOF [s^−1^]
Sil-1	batch reactor	90 °C, 4 h	2.3	0.06
Sil-1_KCl	batch reactor	90 °C, 4 h	7.9	0.2
Sil-1_KF	batch reactor	90 °C, 4 h	9.9	0.3
Sil-1_KOH	batch reactor	90 °C, 4 h	90.9	2.3
Sil-1	batch reactor	70 °C, 15 min	0.7	0.3
Sil-1_KOH	batch reactor	70 °C, 15 min	5.3	2.1
Sil-1	ultrasonic bath	70 °C, 15 min	6.3	2.6
Sil-1_KOH	ultrasonic bath	70 °C, 15 min	13.4	5.4
Sil-1	ultrasonic probe in bath	70 °C, 15 min	18.6	7.5
Sil-1_1KOH	ultrasonic probe in bath	70 °C, 15 min	71.3	28.9
Sil-1	ultrasonic probe in the reaction mixture	70 °C, 15 min	49.4	20.0
Sil-1_1KOH	ultrasonic probe in the reaction mixture	70 °C, 15 min	96.6	39.1

## Data Availability

The data presented in this study are available on request from the corresponding author.

## Supplementary Materials

The following supporting information can be downloaded at https://www.mdpi.com/article/10.3390/molecules30071590/s1; Table S1. Infrared band assignments in silica-based materials. Table S2. Density (µmol/m²) of basic sites with varying strengths in the obtained catalysts, expressed as the number of sites per unit surface area (m^2^).; Figure S1. SEM image of Sil-1_KOH sample. Figure S2. Linear correlation factor as a function of the number of each type of basic site (total, low-temperature, medium-temperature, and high-temperature) with glycerol conversion (red dot- Sil-1, blue dot-Sil-1_KCl, green dot- Sil-1_KF, and black dot- Sil-1_KOH). Figure S3. The relationship between glycerol conversion and concentration of the number of basic sites of different strengths. Figure S4. The relationship between glycerol conversion and glycerol carbonate yield with the density of the total number of basic sites. Figure S5. Effect of the applied procedure (batch reactor, ultrasonic bath, ultrasonic probe in bath or ultrasonic probe) on TOF using as-synthesized Sil-1 and potassium-modified Sil-1_KOH) (reaction conditions: 10 wt.% of catalysts, glycerol-to-DMC molar ratio of 1:5, 70 °C, 15 min). Scheme S1. The proposed reaction pathway for the synthesis of glycerol carbonate from glycerol and dimethyl carbonate (DMC) on potassium-modified Silicalite-1. Scheme S2. Different methods applied in the transesterification of glycerol with DMC to obtain glycerol carbonate: ultrasonic probe (A), batch reactor (B), and ultrasonic bath (C). Calculation of the catalytic activity results based on the chromatographic data. Identification of components of the post-reaction mixture using GC-MS analysis. Details of the quantitative analysis of the post-synthesis reaction mixture were performed using gas chromatography [56].
