# Supplementary material for: Ultrasound-Assisted Synthesis of Glycerol Carbonate Using Potassium-Modified Silicalite-1 as a Catalyst
Source: Molecules. 2025 Apr 2;30(7):1590. doi: 10.3390/molecules30071590 (PMC11990769; doi:10.3390/molecules30071590)

SUPPLEMENTARY MATERIAL

Ultrasound-Assisted Synthesis of Glycerol Carbonate Using Potassium-Modified Silicalite-1 as a Catalyst

Jolanta Kowalska-Kuś, Ewa Janiszewska*, Agnieszka Held, Aldona Jankowska, Anetta Hanć, Stanisław Kowalak*

Adam Mickiewicz University in Poznań, Faculty of Chemistry, Uniwersytetu Poznańskiego 8, 61-614 Poznań, Poland

*Corresponding author: Ewa Janiszewska

e-mail: eszym@amu.edu.pl

*Corresponding author: Stanisław Kowalak

e-mail: skowalak@amu.edu.pl

Table S1. Infrared bands assignments in silica-based materials.

Vibrations	Internal tetrahedra [cm^{-1}]	External linkages [cm^{-1}]
ν_{as} (T-O-T)	950-1250	1150-1050
ν_s (T-O)	650-720	750-820
δ (O-T-O) double ring	-	500-650
T-O bend	420-500	-

Jansen, J.C.; van der Gaag, F.J.; van Bekkum H. Identification of ZSM-type and other 5-ring containing zeolites by i.r. spectroscopy. *Zeolites* **1984**, *4 (4)*, 369-372. https://doi.org/10.1016/0144-2449(84)90013-7

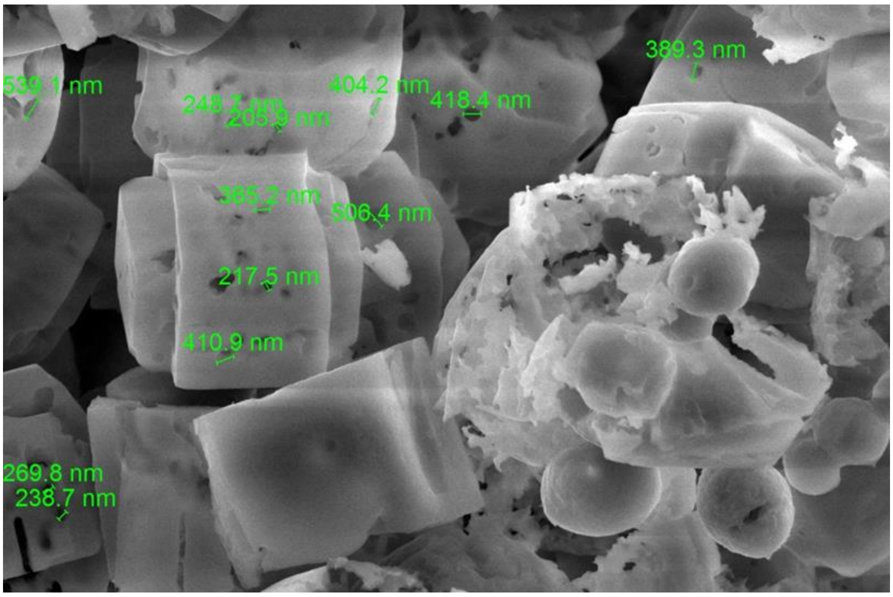

Figure S1. SEM image of Sil-1_KOH sample.

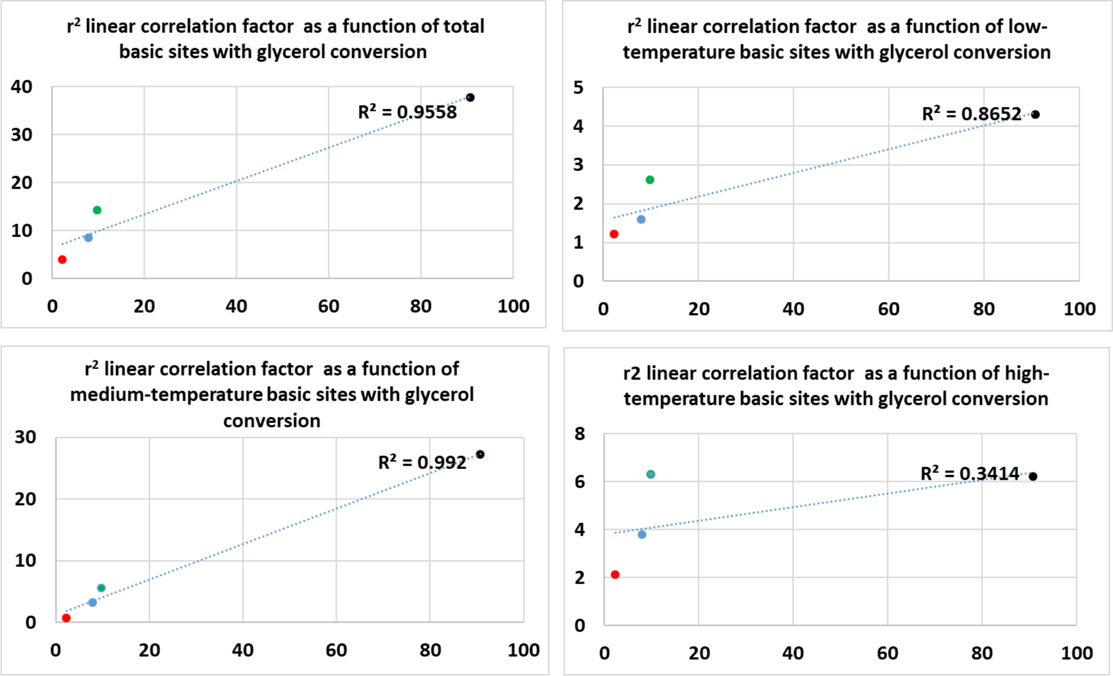

Figure S2. Linear correlation factor as a function of the number of each type of basic site (total, low-temperature, medium-temperature, and high-temperature) with glycerol conversion (red dot- Sil-1, blue dot-Sil-1_KCl, green dot- Sil-1_KF, and black dot- Sil-1_KOH).

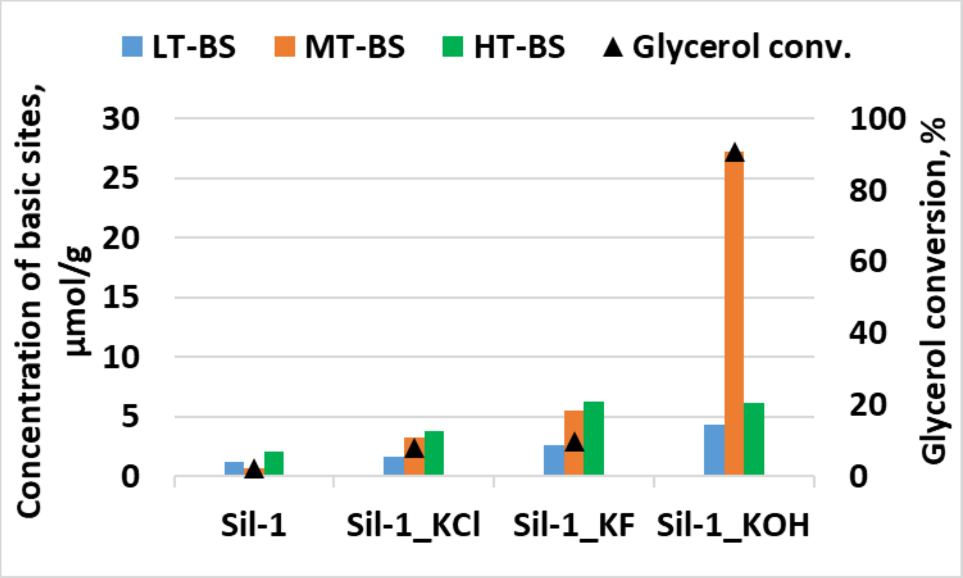

Figure S3. The relationship between glycerol conversion and concentration of the number of basic sites of different strength.

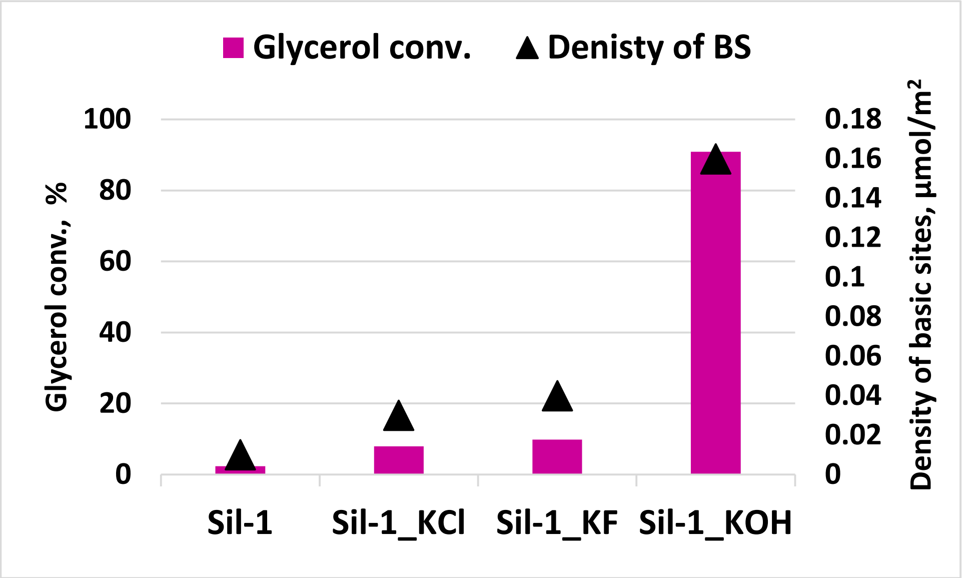

Figure S4. The relationship between glycerol conversion and glycerol carbonate yield with the density of the total number of basic sites.

Table S2. Density (μmol/m²) of basic sites with varying strengths in the obtained catalysts, expressed as the number of sites per unit surface area (m²).

Sample	Density of Basic Sites, μmol/m²			
	LT-BS<br>T ≤ 200 °C	MT-BS<br>200–350 °C	HT-BS<br>T ≥ 350 °C	Total BS/S_{BET}
Sil-1	0.004	0.002	0.007	0.01
Sil-1_1KCl	0.005	0.010	0.012	0.03
Sil-1_1KF	0.008	0.016	0.018	0.04
Sil-1_1KOH	0.019	0.118	0.027	0.16

LT-BS - the number of low-temperature basic site; MT-BS - the number of medium-temperature basic site; HT-BS - the number of high-temperature basic site

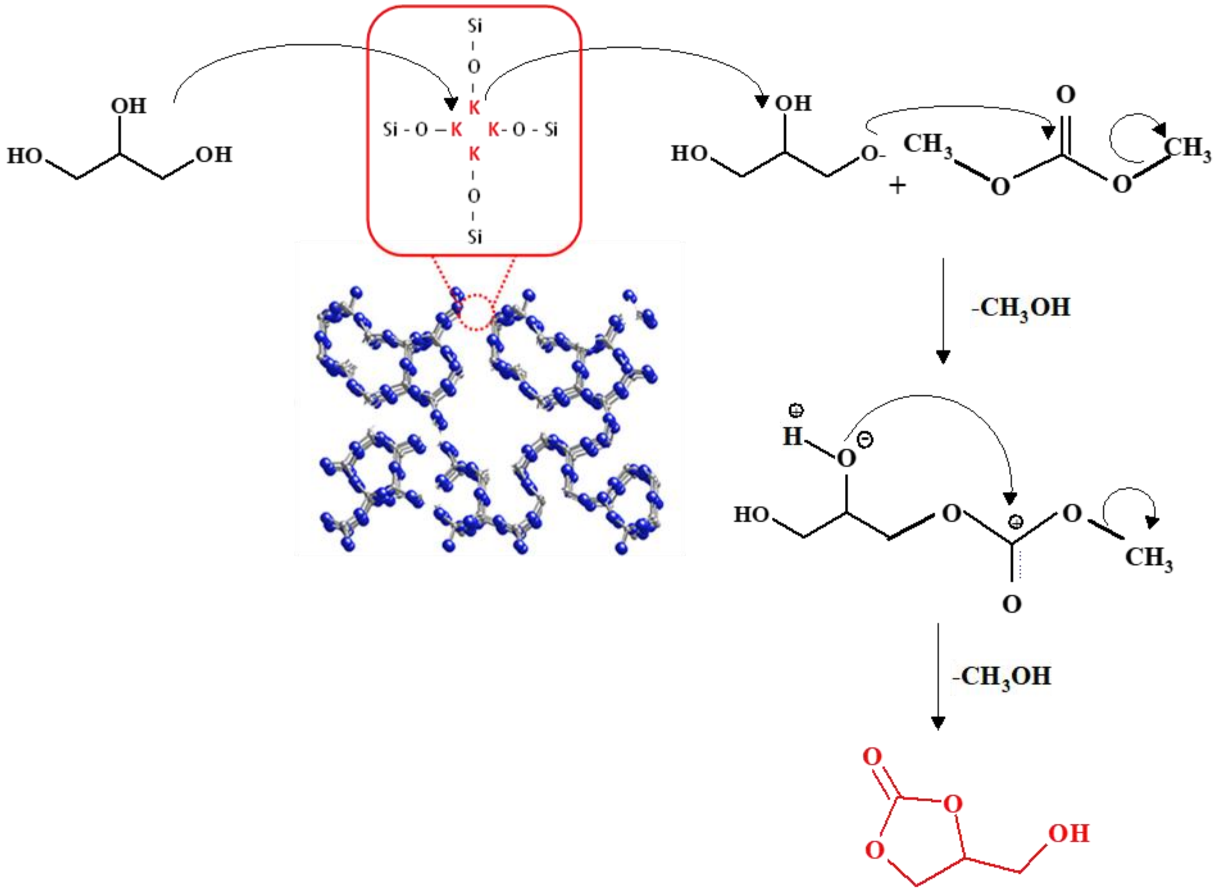

Scheme S1. The proposed reaction pathway for the synthesis of glycerol carbonate from glycerol and dimethyl carbonate (DMC) on potassium-modified Silicalite-1

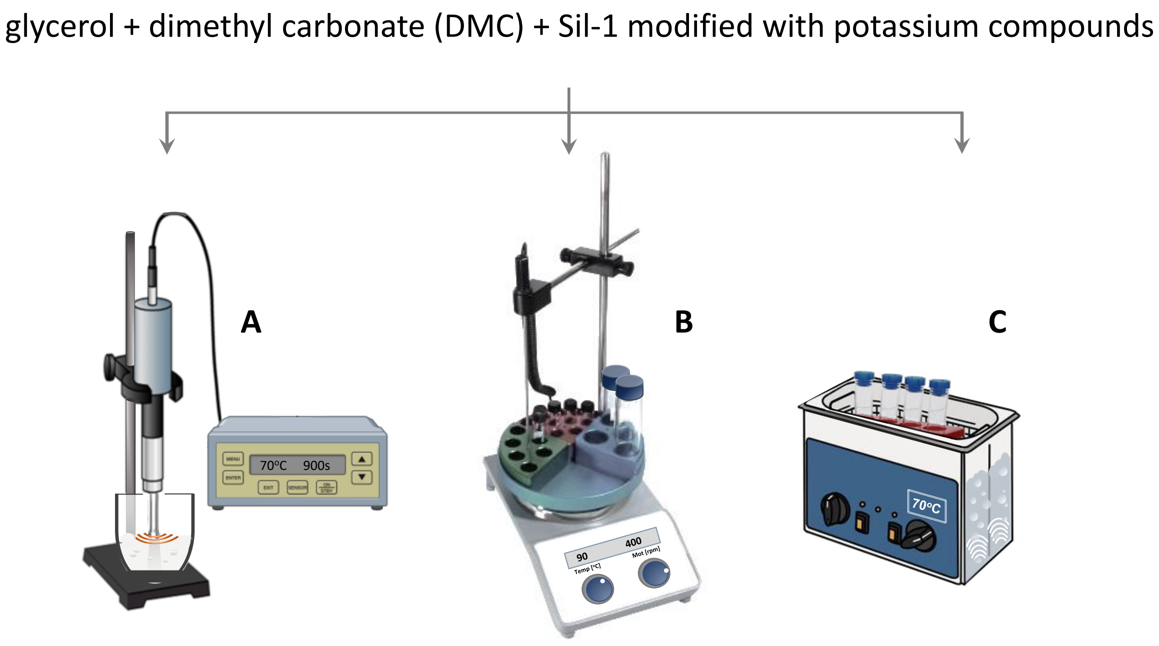

Scheme S2. Different methods applied in the transesterification of glycerol with DMC to obtain glycerol carbonate: ultrasonic probe (A), batch reactor (B), and ultrasonic bath (C).

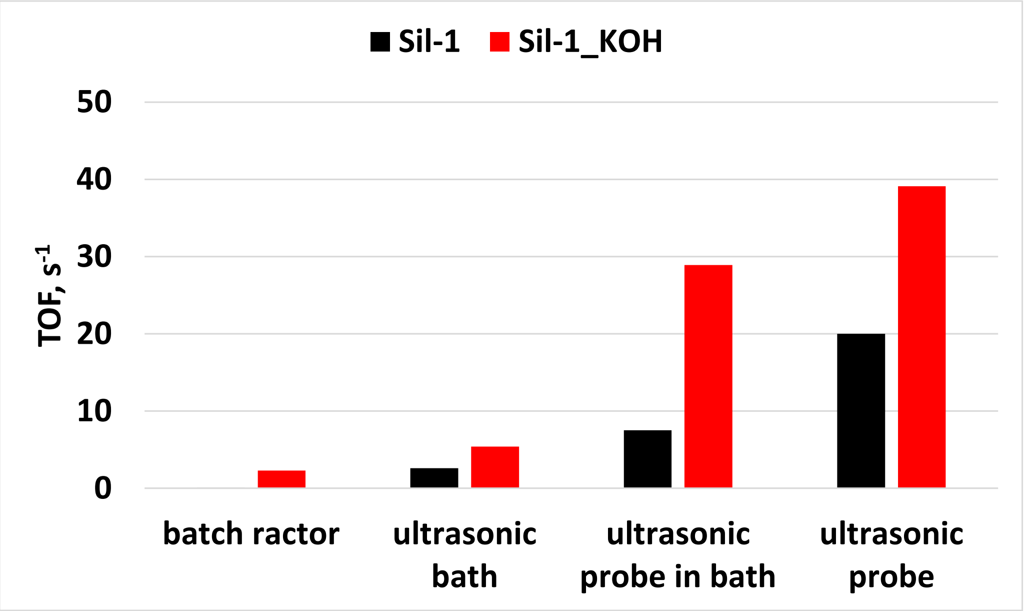

Figure S5. Effect of the applied procedure (batch reactor, ultrasonic bath, ultrasonic probe in bath or ultrasonic probe) on TOF using as synthesized Sil-1 and potassium modified Sil-1_KOH (reaction conditions: 10 wt.% of catalysts, glycerol-to-DMC molar ratio of 1:5, 70 °C, 15 min).

Calculation of the catalytic activity results based on the chromatographic data

Conversion of glycerol (%)

$$Glycerol\ conv. = \frac{moles\ of\ glycerol\ converted}{initial\ moles\ of\ glycerol} \times 100 \qquad \text{(Eq. 1)}$$

where moles of glycerol converted = initial moles of glycerol – final moles of glycerol

Selectivity to glycerol carbonate (%)

$$S\ Glyc.Carbonate = \frac{moles\ of\ glycerol\ carbonate\ formed}{moles\ of\ glycerol\ converted} \times 100 \qquad \text{(Eq. 2)}$$

Yield of glycerol carbonate (%)

$$Y\ Glyc.Carbonate = \frac{S\ glyc.\ carbonate \bullet Glycerol\ conv.}{100} \qquad \text{(Eq. 3)}$$

Identification of components of the post-reaction mixture using GC-MS analysis

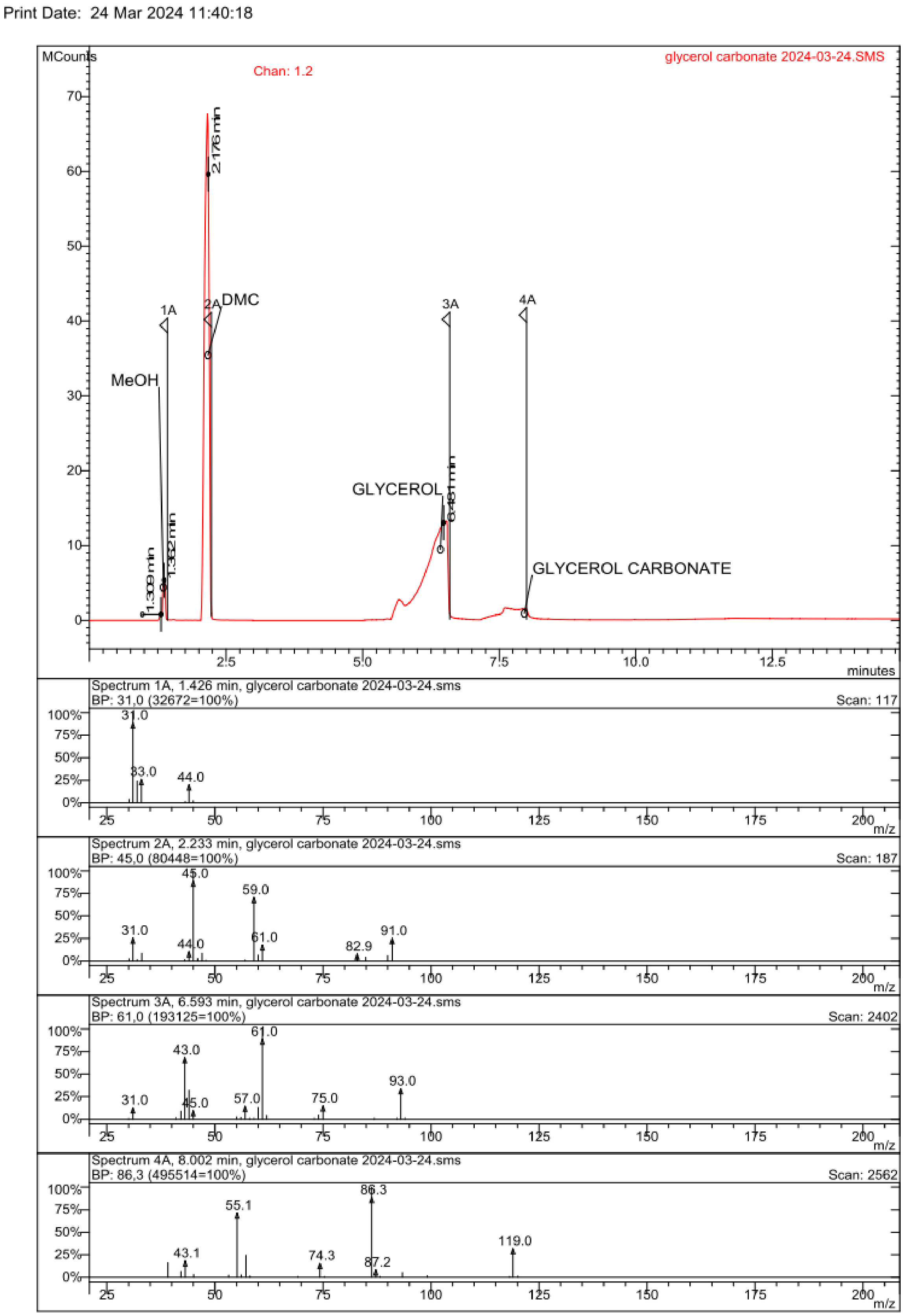

Details of the quantitative analysis of the post-synthesis reaction mixture was performed using gas chromatography

1. **Program used for the analysis of the reaction mixture on the VARIAN CP-3800 chromatograph equipped with a Flame Ionization Detector (FID) and a VF-5ms column**

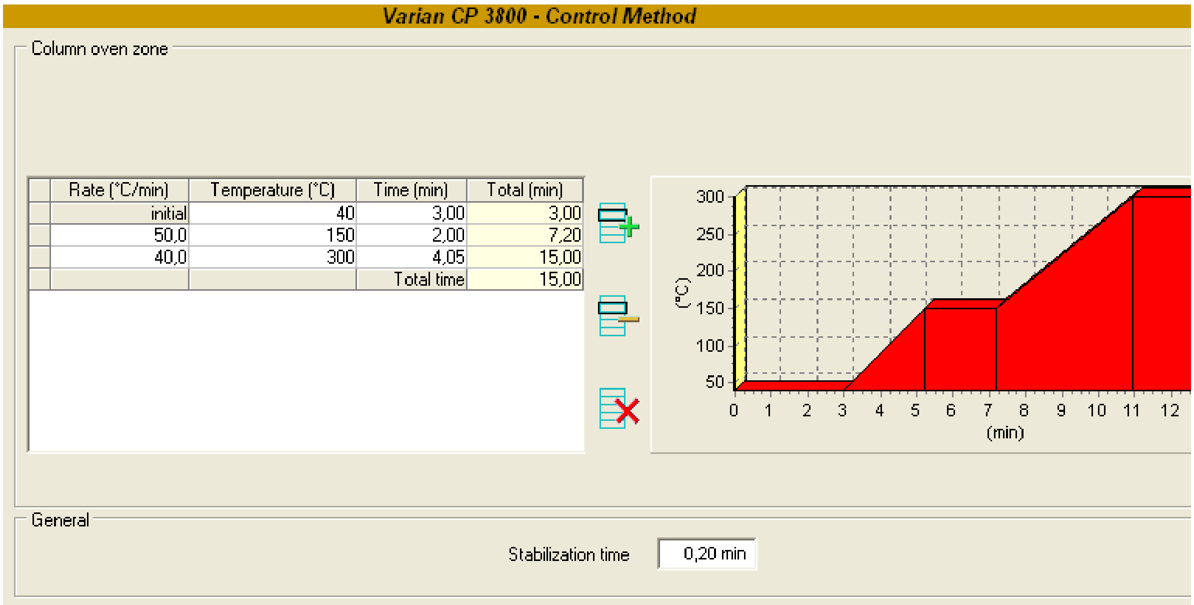

Rate (°C/min)	Temperature (°C)	Time (min)	Total (min)
initial	40	3,00	3,00
50,0	150	2,00	7,20
40,0	300	4,05	15,00
		Total time	15,00

2. **Chromatograms**

Sil-1 batch reactor 90 °C, 4h

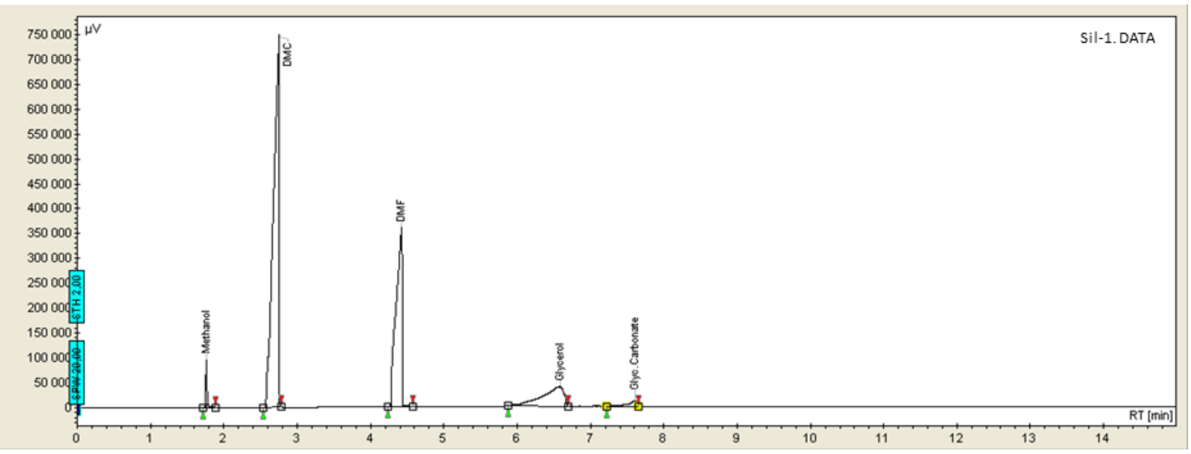

Sil-1_KCl   batch reactor   90 °C, 4h

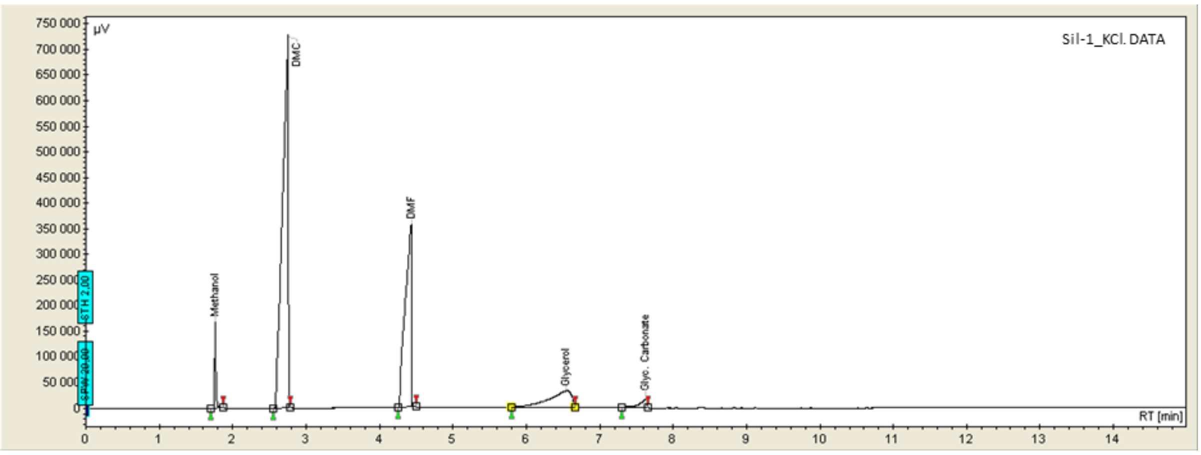

Sil-1_KF   batch reactor   90 °C, 4h

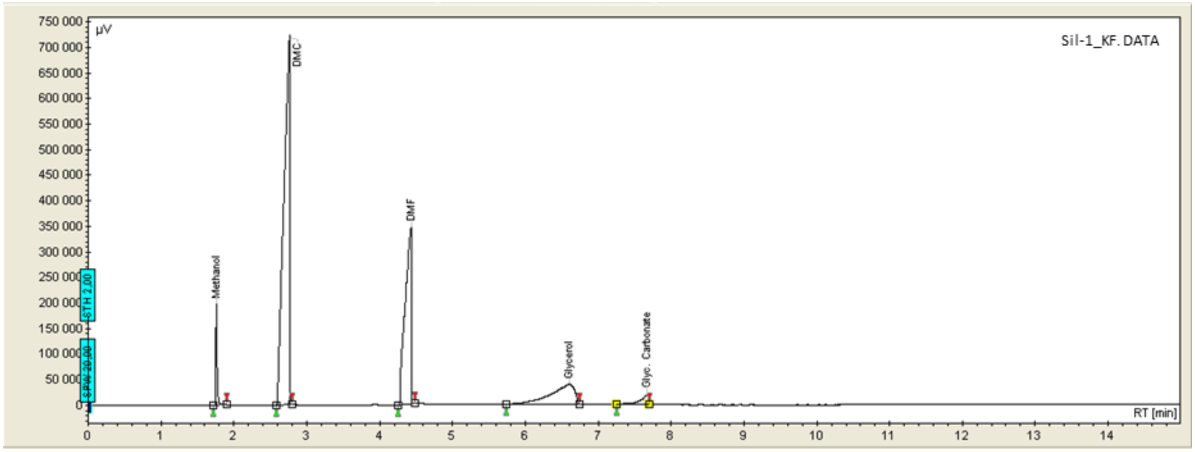

Sil-1_KOH   batch reactor   90 °C, 4h

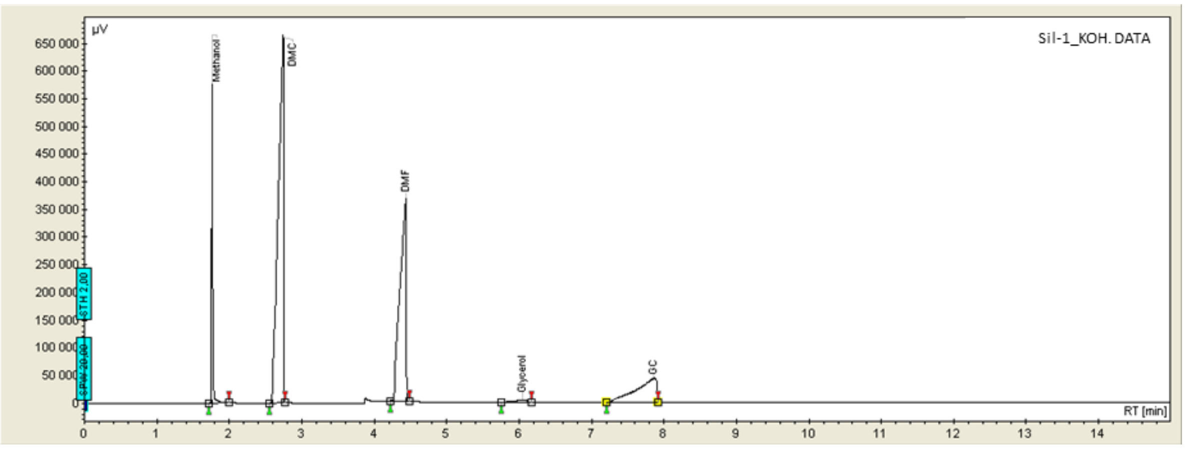

Sil-1 batch reactor 70 °C, 15min

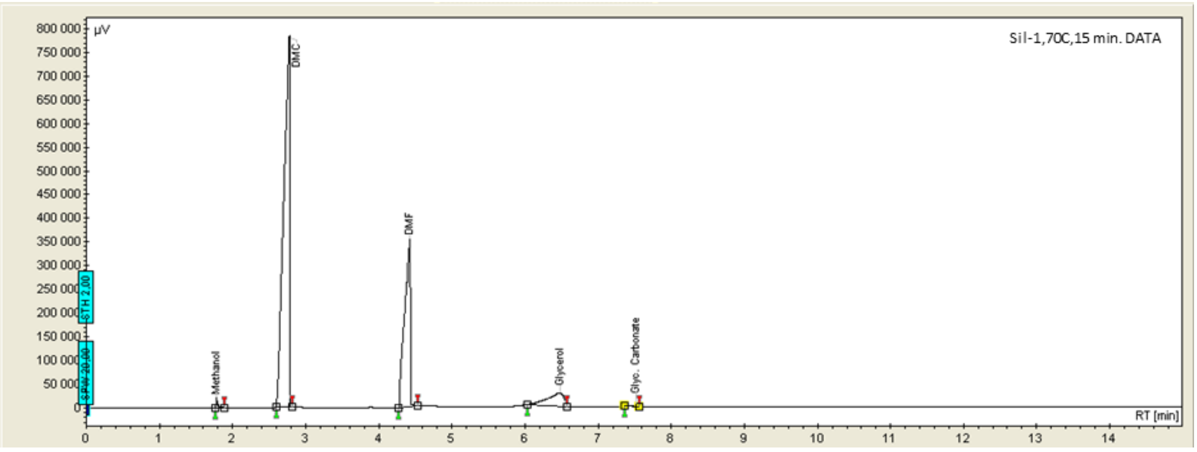

Sil-1_KOH batch reactor 70 °C, 15min

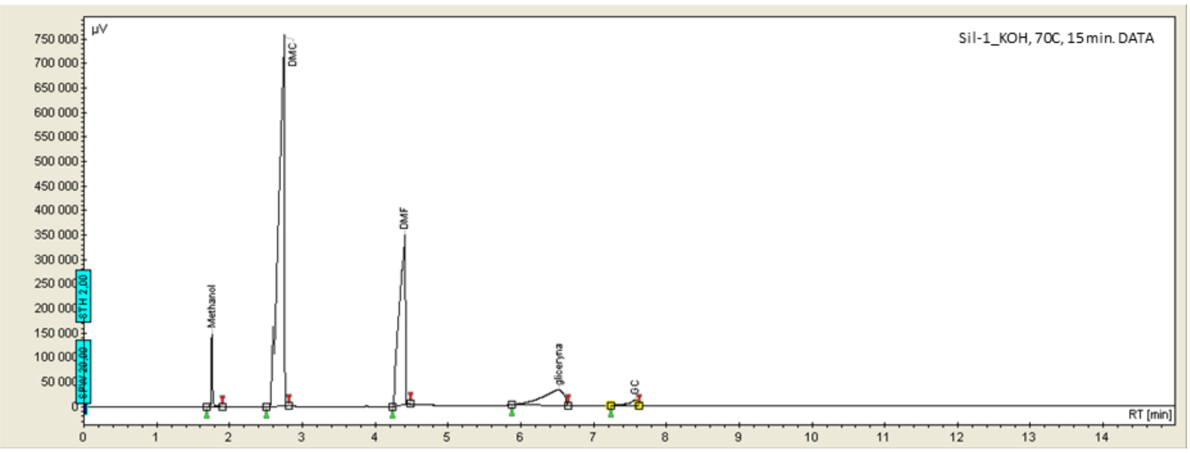

Sil-1 ultrasonic bath 70 °C, 15 min

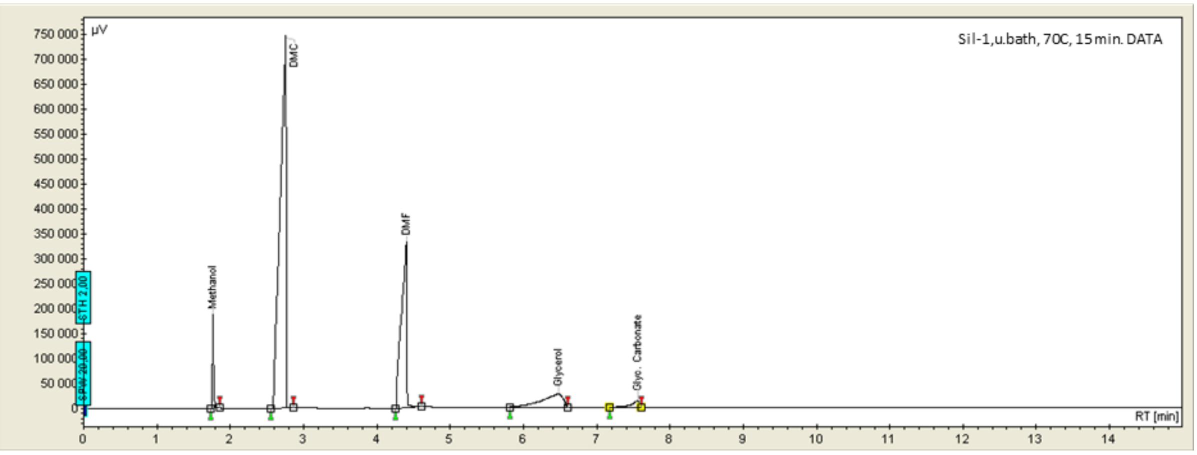

Sil-1_KOH ultrasonic bath 70 °C, 15 min

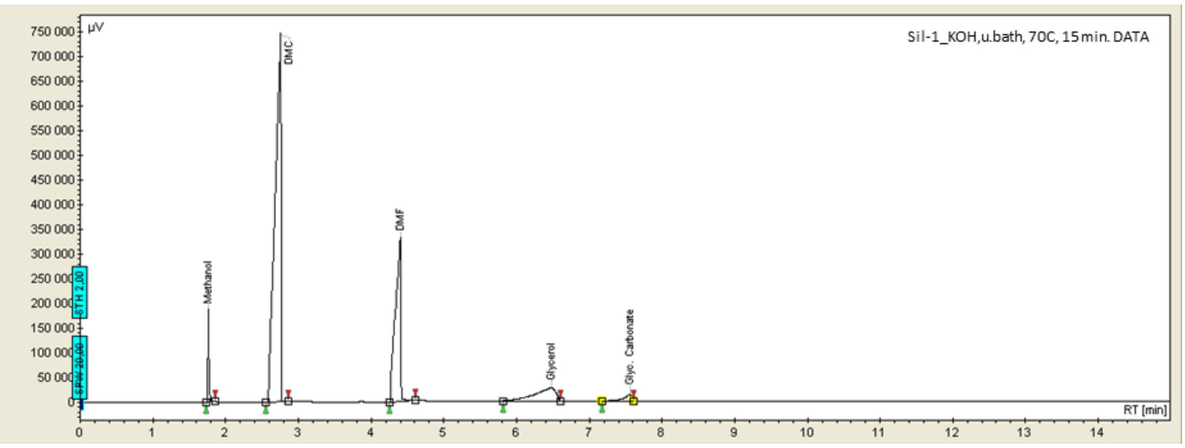

Sil-1 ultrasonic probe in bath 70 °C, 15 min

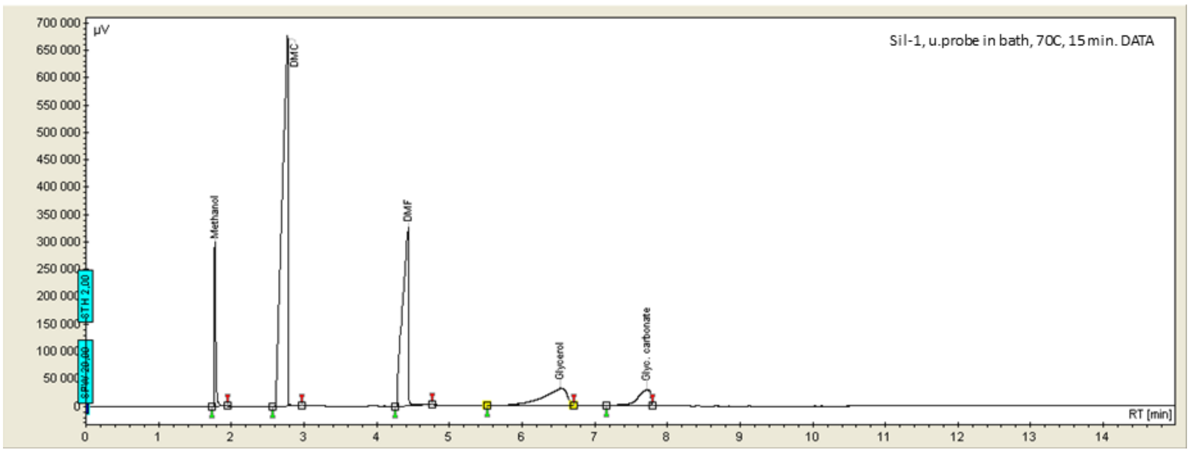

Sil-1_KOH ultrasonic probe in bath 70 °C, 15 min

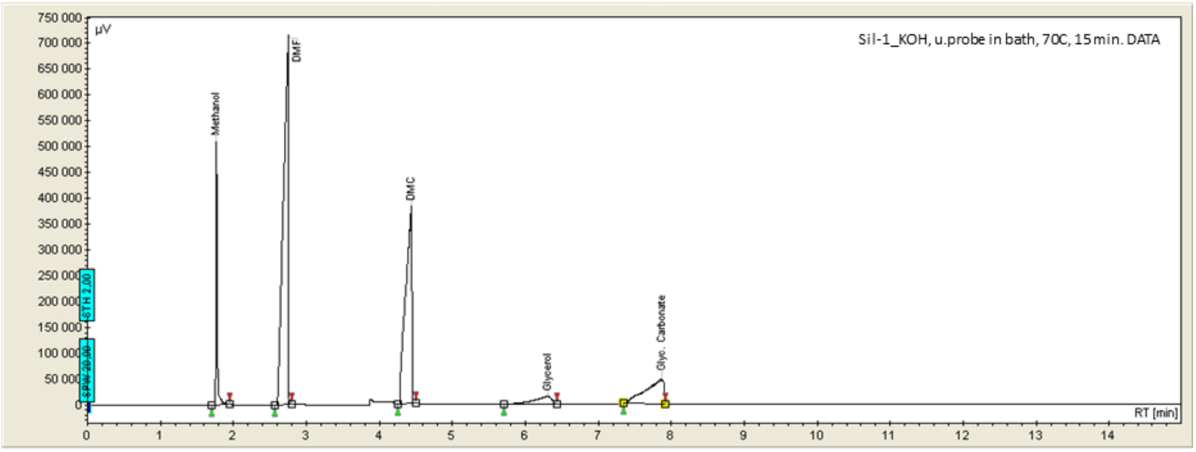

Sil-1 ultrasonic probe in the reaction mixture 70 °C, 15 min

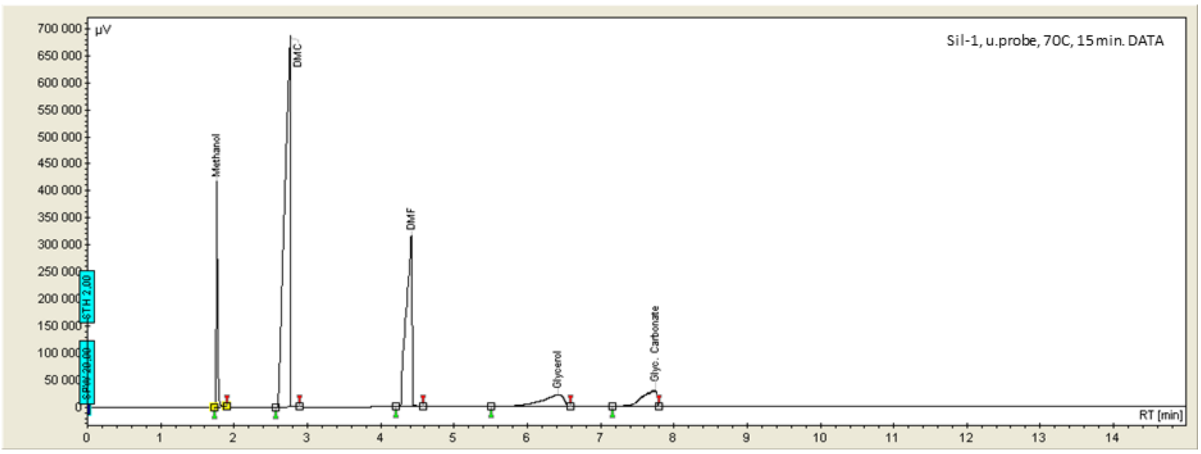

Sil-1_KOH ultrasonic probe in the reaction mixture 70 °C, 15 min

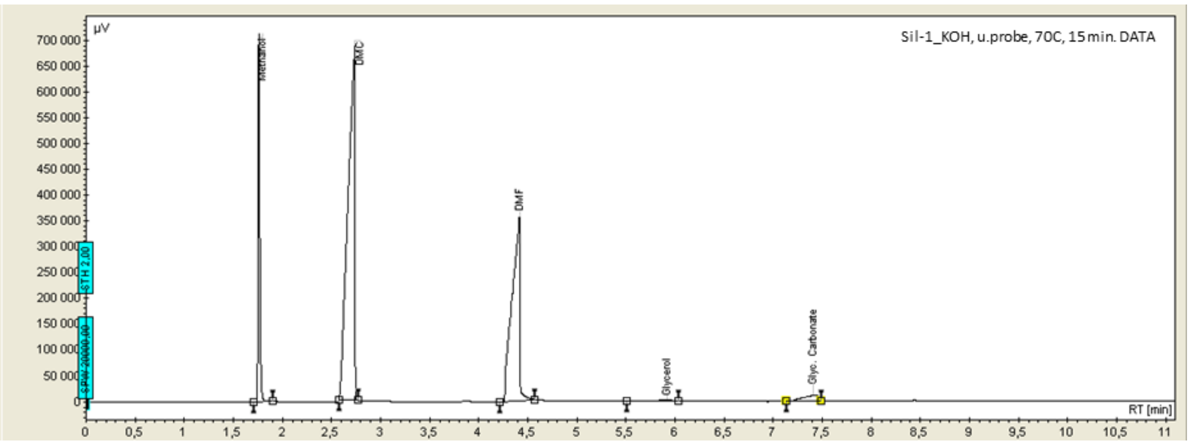

Supplement: Supplementary file 1 [file molecules-30-01590-s001.zip › molecules-3529407-supplementary.pdf]
